# Supplementary material for: Understanding money-management behaviour and its potential determinants among undergraduate students: A scoping review
Source: PLoS One. 2024 Aug 15;19(8):e0307137. doi: 10.1371/journal.pone.0307137 (PMC11326551; doi:10.1371/journal.pone.0307137)
Supplement: S1 Table — (DOCX) [file pone.0307137.s001.docx]

**Supplementary Table. Characteristics of reviewed studies**

| *Reference* | *Sample & setting* | *Study type* | *Behaviours studied* | *Psychological theory used? (If yes, which?)* | *Quantitative data only:*  *Psychological variables studied* |
| --- | --- | --- | --- | --- | --- |
| Bamforth & Geursen (2017)  +  Bamforth et al (2017) | Full-time undergraduates  N = 40 (24 females, 16 males)  Age range 18-24y  Australia | Qualitative-only | *Borrowing*  - Using credit cards | No | N/A |
| Chan et al (2012) | University students  N = 821 (67% female, 34% male)  Age range 18-30y  Mean age 21.1 years  Hong Kong | Quantitative-only | *Budgeting*  - Tracking expenses  - Comparing prices  - Planning finance  *Borrowing*  - Using credit card  *Settling debts*  - Making minimal payments on loans | - Money Management Model | *Personality characteristics*  *-* Impulsivity  *Attitudes*  *-* Anticipated income  - Attitudes towards debt  *Affective responses*  - Financial wellbeing  - Emotional evaluation - Anxiety  *Self-efficacy and control*  - Perceived control |
| Johan et al (2021) | Undergraduate students  N = 521 (307 female, 214 male)  Age range 17-24y  Indonesia | Mixed-method | *Borrowing*  - Using credit cards  *Budgeting*  - Creating or maintaining a budget  - Tracking expenses  - Comparing prices | - Consumer Socialisation Theory | *Financial beliefs and knowledge*  - Financial literacy  *Social influence*  - Communication with parents |
| Kidwell et al (2003) | Psychology students  N = 250 (136 female, 114 male)  (Age not reported)  USA | Quantitative-only | *Budgeting*  - Creating or maintaining a budget | - Theory of Reasoned Action  - Theory of Interpersonal Behaviour | *Attitude*  - Attitude towards budgeting  *Affective responses*  - Budgeting affect  *Self-efficacy and control*  - Perceived behaviour  - Perceived ability |
| McClure & Ryder (2018) | College students  N = 426 (quantitative component; 86% female, 14% male)  N = 28 (qualitative)  (Age not reported)  USA | Mixed-method | *Spending*  - Overspending | No | *Social influences*  *-* Peer influence |
| Sachitra et al (2019) | Undergraduate students  N = 40 (focus groups; 22 females, 18 males)  N = 13 (interviews; 7 females; 6 males)  (Age not reported)  Sri Lanka | Qualitative-only | *Budgeting*  - Creating or maintaining a budget  *Settling debts*  - Making credit card payments in full  *Spending*  Saving (minimising usual expenditure)  *Saving (building funds)*  - Accruing money | No | N/A |
| Sages et al (2013) | Students who had sought financial counselling  N = 307 (61% female, 39% male)  (Age not reported)  USA | Quantitative-only | *Borrowing*  - Use of cash advance on credit card  - Maximising credit card  *Settling debts*  - Settling debts on time  *Spending*  - Overspending | No | *Affective responses*  - Anxiety |
| Shim et al (2010)  +  Xiao et al (2011) | First-year undergraduates  N = 2098 (Shim et al, 2010; 62% female)  N = 1242 (Xiao et al, 2011; subsample who own credit card; 60% female)  (Age not reported)  USA | Quantitative-only | *Borrowing*  - Using credit cards  - Maximising credit cards  *Budgeting*  - Tracking expenses  *Settling debts*  - Making credit card payments in full  *Spending*  *-* Overspending  *Saving (building funds)*  - Accruing money  - Investing | - Theory of Planned Behaviour (Xiao et al, 2011) | *Financial beliefs and knowledge*  - Financial knowledge  - Parental direct teaching  *Attitude*  - Financial attitude  *Self-efficacy and control*  - Perceived financial behaviour  *Social influences*  - Parental descriptive norms  - Parental subjective norms  - Parental relationship |
| Sundarasan et al (2016) | Undergraduate students  N = 200 (60% female, 40% male)  Age range 20-40y  Malaysia | Quantitative-only | *Settling debts*  *-* Making credit card payments in full  *Saving (building funds)*  - Accruing money | No | *Social influences*  - Parental norm  - People and media  *Financial beliefs and knowledge*  - Financial literacy  - Financial knowledge |
| Yanto et al (2021) | Economics and business students  N = 327  (Gender and age not reported)  Indonesia | Quantitative-only | *Budgeting*  *Settling debts*  *Saving (building funds)*  - No specific behaviours identified | No | *Attitudes*  *-* Financial attitude  *Social influences*  - Social media exposure  - Peer influence |
