# Supplementary material for: Understanding money-management behaviour and its potential determinants among undergraduate students: A scoping review
Source: PLoS One. 2024 Aug 15;19(8):e0307137. doi: 10.1371/journal.pone.0307137 (PMC11326551; doi:10.1371/journal.pone.0307137)
Supplement: Attachment — Submitted filename: Review_24_18530.pdf [file pone.0307137.s002.pdf]

Review

Understanding money-management behaviour and its potential determinants among undergraduate students: A scoping review

Manuscript Number: PONE-D-24_18530

Author: Benjamin Gardner

The research deals with a relevant topic, its examination contributes significantly to the understanding of university students' money management behaviour and its possible determinants.

The article adequately reviews and contextualizes the examined problem. The adapted method and the interpretation of the results are appropriate. The content is concisely described and contextualized with previous and current theoretical background and empirical research on the topic.

I propose a minor revision in the literature review. The authors did not refer to sources that were pioneers in the research of the topic, e.g.

Bakken, M.R. (1966). Money Management Understanding of Tenth Grade Students. University of Alberta. https://archive.org/details/Bakken1966/page/n13/mode/2up

Chen, H., & Volpe, R.P. (1998). An Analysis of Personal Financial Literacy Among College Students. Financial Services Review, 7(2), 107–128. https://doi.org/10.1016/s1057-0810(99)80006-7

Chen, H., & Volpe, R.P. (2002). Gender differences in personal financial literacy among college students. Financial Services Review, 11(3), 289–308. https://go.gale.com/ps/i.do?p=AONE&sw=w&issn=10570810&v=2.1&it=r&id=GALE%7CA149166047&sid=googleScholar&linkaccess=abs

Danes, S.M., & Hira, T.K. (1987). Money Management Knowledge of College Students. Journal of Student Financial Aid, 17(1), 4–16.

May I suggest a brief explanation of whether your research is relevant only in the UK, or whether it presents a similar problem in another country or continent? The focus of their research may thus change, since it is possible that behaviour related to money management is not only a problem related to higher education.

I also recommend thinking about whether the revealed "problem" as financial pressure really existed from the 1980s to 2023, which is the time when the sampling studies were made.

Based on their methodology, I would like to explain why only 12 of the 789 identified studies met the selection criteria. It is recommended to think about the possibility of conducting the research in other languages (not only English) and involving the appropriate studies in their research. The revealed correlates and categories can form the basis of empirical research in the future.

I also recommend a more detailed discussion of the problem that the parental model and the opinion of peers have a positive effect on the financial behaviour of university students, because according to European research experience, the behaviour and opinion of parents and peers have a negative effect on the appropriate financial behaviour. This is confirmed by e.g. also lines 285-287.

After these changes, I recommend the article for acceptance.
